# Applying a polysaccharide lyase from *Stenotrophomonas maltophilia* to disrupt alginate exopolysaccharide produced by *Pseudomonas aeruginosa* clinical isolates

**DOI:** 10.1128/aem.01853-24

**Published:** 2024-12-13

**Authors:** Samantha M. Felton, Nikki Akula, Glynis L. Kolling, Parastoo Azadi, Ian Black, Ambrish Kumar, Christian Heiss, Joseph Capobianco, Joseph Uknalis, Jason A. Papin, Bryan W. Berger

**Affiliations:** 1 Department of Biomedical Engineering, University of Virginia 174483 , Charlottesville, Virginia, USA; 2 Complex Carbohydrate Research Center, University of Georgia 123423 , Athens, Georgia, USA; 3 United States Department of Agriculture (USDA), Agricultural Research Service (ARS), Eastern Regional Research Center 57694 , Wyndmoor, Pennsylvania, USA; 4 Department of Chemical Engineering, University of Virginia 2358 , Charlottesville, Virginia, USA; Shanghai Jiao Tong University, Shanghai, China

**Keywords:** exopolysaccharide, alginate, *Pseudomonas aeruginosa*, *Stenotrophomonas*, mucoid, alginate lyase

## Abstract

*Pseudomonas aeruginosa* is considered one of the most challenging, drug-resistant, opportunistic pathogens partly due to its ability to synthesize robust biofilms. Biofilm is a mixture of extracellular polymeric substances (EPS) that encapsulates microbial cells, leading to immune evasion, antibiotic resistance, and thus higher risk of infection. In the cystic fibrosis lung environment, *P. aeruginosa* undergoes a mucoid transition, defined by overproduction of the exopolysaccharide alginate. Alginate encapsulation results in bacterial resistance to antibiotics and the host immune system. Given its role in airway inflammation and chronic infection, alginate is an obvious target to improve treatment for *P. aeruginosa* infection. Previously, we demonstrated polysaccharide lyase Smlt1473 from *Stenotrophomonas maltophilia* strain k279a can catalyze the degradation of multiple polyuronides *in vitro*, including D-mannuronic acid (poly-ManA). Poly-ManA is a major constituent of *P. aeruginosa* alginate, suggesting that Smlt1473 could have potential application against multidrug-resistant *P. aeruginosa* and perhaps other microbes with related biofilm composition. In this study, we demonstrate that Smlt1473 can inhibit and degrade alginate from *P. aeruginosa*. Additionally, we show that tested *P. aeruginosa* strains are dominant in acetylated alginate and that all but one have similar M-to-G ratios. These results indicate that variation in enzyme efficacy among the isolates is not primarily due to differences in total EPS or alginate chemical composition. Overall, these results demonstrate Smlt1473 can inhibit and degrade *P. aeruginosa* alginate and suggest that other factors including rate of EPS production, alginate sequence/chain length, or non-EPS components may explain differences in enzyme efficacy.

**IMPORTANCE:**

*Pseudomonas aeruginosa* is a major opportunistic human pathogen in part due to its ability to synthesize biofilms that confer antibiotic resistance. Biofilm is a mixture of polysaccharides, DNA, and proteins that encapsulate cells, protecting them from antibiotics, disinfectants, and other cleaning agents. Due to its ability to increase antibiotic and immune resistance, the exopolysaccharide alginate plays a large role in airway inflammation and chronic *P. aeruginosa* infection. As a result, colonization with *P. aeruginosa* is the leading cause of morbidity and mortality in CF patients. Thus, it is an obvious target to improve the treatment regimen for *P. aeruginosa* infection. In this study, we demonstrate that polysaccharide lyase, Smlt1473, inhibits alginate secretion and degrades established alginate from a variety of mucoid *P. aeruginosa* clinical isolates. Additionally, Smlt1473 differs from other alginate lyases in that it is active against acetylated alginate, which is secreted during chronic lung infection. These results suggest that Smlt1473 may be useful in treating infections associated with alginate-producing *P. aeruginosa*, as well as have the potential to reduce *P. aeruginosa* EPS in non-clinical settings.

## INTRODUCTION

Microbial cells can exist in a tightly linked network known as a biofilm (1). Biofilms are formed in a multi-stage process when bacterial cells colonize a surface via adhesion, followed by encapsulation in an extracellular polymeric substance (EPS) matrix comprised of polysaccharides, proteins, cell debris, and nucleic acids (2). Reversible attachment occurs initially, followed by EPS production to form an adherent state (1). Of note, in some cases, biofilm can form without surface attachment (3). As the EPS accumulates, the biofilm matures, and microcolonies detach from the biofilm and colonize other surfaces or solutions (4). A mature biofilm provides a valuable defense by acting as a barrier limiting antibiotic penetration and shielding cells from immune system detection, thereby reducing opsonization (5, 6). Furthermore, detoxification enzymes are often present within *Pseudomonas aeruginosa* biofilm EPS. One example is the major catalase, KatA, in *P. aeruginosa*, which offers additional protection to the cell to survive under stress conditions (7, 8). Thus, there is a need for novel approaches to inhibit and remove microbial biofilms in order to reduce pathogen persistence, spread, and antibiotic resistance.

*P. aeruginosa* is one of the most frequently occurring opportunistic pathogens responsible for hospital-acquired infections. Its ability to form biofilms is a major factor contributing to its widespread hospital occurrence (9). Moreover, *P. aeruginosa* is the primary pathogen associated with drug-resistant infections in patients with cystic fibrosis (CF). In patients with CF, the mucoid phenotype, characterized by the overproduction of the exopolysaccharide alginate, accelerates a decline in lung function (10, 11). Alginate is a heteropolymer comprised of two uronic acids, mannuronic acid (M) and guluronic acid (G). Differences in the M-to-G ratio in alginate confer changes in biofilm mechanical properties, as well as metal and antibiotic binding abilities (12–15). For example, increases in mannuronic acid content have been shown to reduce biofilm permeability to antibiotics (16). *P. aeruginosa*-synthesized alginate can also be acetylated, which has been implicated in enhancing resistance to phagocytosis, increasing biofilm viscosity and hydrophobicity, and creating 3D biofilm architectures (12, 17, 18). Thus, the properties of mucoid biofilms are strongly dependent on alginate chemical composition, and thus, *P. aeruginosa* biofilm removal strategies must be able to address a range of alginate compositions and properties.

Enzymes are a promising approach to targeting and degrading specific biofilm components including EPS and DNA (19). Enzymes also hold promise for synergizing with antimicrobial agents and photodynamic therapy methods to treat biofilm-positive infections more effectively. Additionally, enzymes may enhance biocompatibility in these approaches (19–21). Alginate lyases are a class of enzymes that degrade uronic acid-containing EPS such as alginate through a β-elimination mechanism. *P. aeruginosa* produces an intracellular alginate lyase (AlgL) that plays a role in regulating alginate secretion during biofilm formation (22). Previous research has investigated AlgL, and other alginate lyases have been explored for mucoid biofilm removal. However, varying effects on alginate biofilm removal are observed depending on the enzyme chosen, leading to variability in antibiotic susceptibility. This is due in part to differences in specificity for M versus G linkages (23). Combinations of enzymes with diverse substrate specificities are often used for removing mucoid biofilms, but typically, testing is limited to reference mucoid strains rather than clinical isolates (23). Thus, a clear relationship between alginate lyase substrate specificity, biofilm composition, and effects on removal, particularly from clinical samples, remains an area of active research.

We previously demonstrated that a predicted alginate lyase, Smlt1473, from *Stenotrophomonas maltophilia* clinical strain k279a, is secreted into the culture, where it can depolymerize multiple substrates including alginate and hyaluronic acid (24). Polysaccharide lyases (PLs) such as Smlt1473 depolymerize uronic acid-containing substrates according to a β-elimination mechanism. Specifically, Smlt1473 belongs to polysaccharide lyase family 5 (PL-5) in the Carbohydrate-Active enzymes (CAZY) database (25). Alginate lyases belonging to the PL-5 family, such as Smlt1473, are known to have specificity towards mannuronic acid (M), in contrast to those in the PL-7 family that generally prefer guluronic acid (G) (26, 27). Given that our carbohydrate composition analysis and NMR data on the polysaccharide fractions from mucoid *P. aeruginosa* clinical isolates show dominant M-rich alginate composition, this characteristic points to PL-5 family lyases with specificity toward M-blocks as prime candidates to target *P. aeruginosa* alginate in the clinical context. A comprehensive study conducted by Blanco-Cabra et al. compared five different alginate lyases against *P. aeruginosa* biofilm; however, four out of the five belonged to the PL-7 family (23). Additionally, Mahajan et al. found four alginate lyases that inhibited *P. aeruginosa* biofilm formation, but again, only one belonged to the PL-5 family (28). Overall, many of the alginate lyases being studied against *P. aeruginosa* belong to the PL-7 family, and the literature contains fewer studies on PL-5 lyases (23, 26, 28, 29). Thus, this study further evaluates the activity of Smlt1473, a member of the less studied PL-5 family, against *P. aeruginosa* alginate. Unique to Smlt1473, the optimal activity toward each substrate is strongly pH-dependent; alginate and poly-mannuronic acid are favored at basic pH (> 7), poly-glucuronic acid is favored over a broad pH range (5–9), and hyaluronic acid is favored at acidic pH (< 7) (24). We also previously solved a high-resolution structure of Smlt1473 in a complex with multiple substrates and provided a mechanism for Smlt1473 in which regions of high dynamic flexibility change with pH to accommodate the chemical diversity of substrates (30).

In this study, we evaluated the ability of Smlt1473 to degrade alginate produced by multiple, clinical isolates of *P. aeruginosa*. Our results demonstrate that Smlt1473 can prevent the formation of alginate as well as remove established alginate across multiple *P. aeruginosa* clinical isolates. However, the concentration of enzyme necessary for alginate inhibition and removal varies across isolates. To understand differences in enzyme concentration needed for efficacy against individual isolates, we characterized the total polysaccharide composition as well as the chemical composition of alginate present in each mucoid isolate. Interestingly, we find that all secreted fractions are essentially acetylated alginate, and the M-to-G ratio of alginate for each is similar across all but one clinical isolate. Thus, the chemical composition of alginate alone does not explain the differences in Smlt1473 concentration needed for EPS removal and inhibition across strains. Addition of Smlt1473 did not inhibit *P. aeruginosa* growth in culture, consistent with its primary effect being on alginate exopolysaccharide rather than on cell wall or surface polysaccharides. Collectively, our results demonstrate that Smlt1473 has a robust ability to inhibit and degrade alginate exopolysaccharide from a range of *P. aeruginosa* clinical isolates. However, through characterization of alginate chemical composition, data suggest that other factors such as the rate of alginate production or percentage of non-EPS components may explain isolate-specific differences in enzyme concentration needed for treatment.

## RESULTS

### Characteristics of clinical isolates and engineered strain

We used multiple mucoid, clinical *P. aeruginosa* isolates in this study to capture a range of potential phenotypes. The strains used, including clinical metadata, are summarized in Table 1. Of particular note, the clinical isolates used include male and female patients over a range of ages and also include patients with CF and diabetes. Finally, the site of isolation for each strain is included. Strain PDO300 is an engineered strain derived from PAO1, which confers a mucoid phenotype and serves as a useful benchmark for Smlt1473 activity against a well-defined mucoid strain (31).

**TABLE 1:** Summary of clinical isolates of *P. aeruginosa* from the UVA Health System Clinical Microbiology Laboratory and engineered strain used in this study

Isolate	Sex	Age	Cystic fibrosis status	Diabetes status	Site of isolation	Extra info
PDO300	NA^*a*^	NA	NA	NA	NA	Derivative of PAO1 with mucA22 allele
UVA 44618	Male	28	Yes	No	Lung/trachea	NA
UVA 55009	Female	39	Yes	Yes	Lung/trachea	NA
UVA 61605	Male	53	No	Yes	Lung/trachea	NA
UVA 84977	Male	20	Yes	No	ENT/sinus	NA

^*a*^ NA, not applicable.

### Smlt1473 inhibits *P. aeruginosa* mucoid phenotype

**Fig 1:**
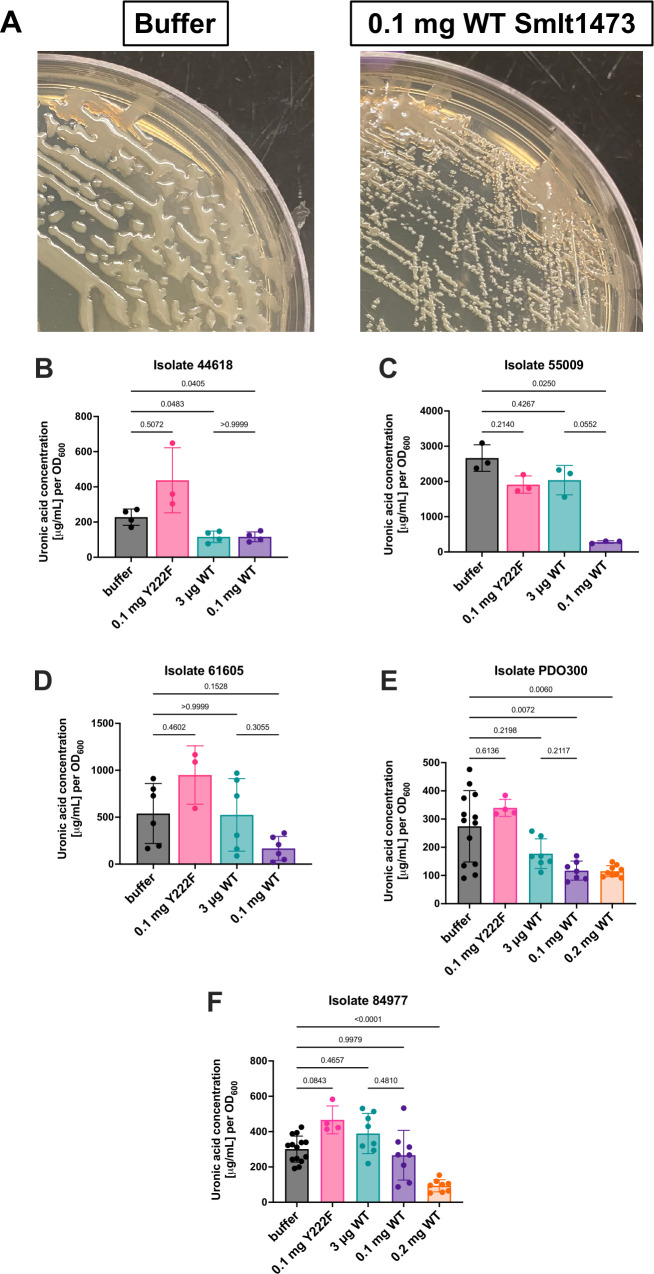
Smlt1473 inhibits the mucoid phenotype of *P. aeruginosa*, but data suggest most effective enzyme concentration varies by isolate. (**A**) Representative image of phenotypic changes for *P. aeruginosa* UVA 44618 in the presence and absence of Smlt1473 indicating an inhibitory function. (**B–F**) Each isolate of *P. aeruginosa* was grown in the presence and absence of Smlt1473, plate contents were collected, and uronic acid concentration, which corresponds to alginate content, was quantified. Uronic acid concentration was determined by measuring the absorbance at 530 nm (A530) of the resulting solution. High A530 corresponds to high alginate content, whereas low A530 corresponds to low alginate content. Y222F is the catalytically inactive form of Smlt1473 used to show that results are due to an active enzyme. The results are means and standard deviations and statistical analysis was performed using a One-way Welch’s ANOVA and Dunnett’s T3 multiple comparison post-hoc test.

To determine if Smlt1473 could inhibit the formation of *P. aeruginosa* mucoid phenotype due to depolymerization of alginate, we coated agar plates with WT Smlt1473 at 0.2 mg, 0.1 mg, and 3 μg concentration and examined the resulting EPS formation for the mucoid isolates by measuring uronic acid concentration, which correlates to alginate content. For clarity, a decrease in the y-axis value correlates to an increase in enzyme activity for inhibition studies, resulting in lower amounts of uronic acid content. Visually, Fig. 1A shows that pre-treatment with 0.1 mg Smlt1473 results in decreased mucoid phenotype for UVA 44618. In the treated condition, there is a decrease in the raised, mucoid architecture, along with increased visibility of single colonies compared with the untreated control. Figure 1B quantifies uronic acid concentration after various treatments for one clinical isolate (UVA 44618) and demonstrates that pre-treatment with both 0.1 mg and 3 μg WT Smlt1473 inhibits the mucoid phenotype for this isolate compared with the untreated control. Additionally, of note, 0.1 mg (high) and 3 μg (low) WT Smlt1473 inhibit the mucoid phenotype of UVA 44618 with near identical efficacy. Figure 1C and D show that uronic acid concentration for two other clinical isolates (UVA 55009 and UVA 61605) is decreased compared with buffer at 0.1 mg Smlt1473 concentration, but not at the 3 μg concentration. These data indicate that inhibiting the mucoid phenotype of different isolates is enzyme-concentration dependent. To demonstrate that the inhibition of the mucoid phenotype is due to Smlt1473, we compared WT results against results using a catalytically inactive mutant (Y222F) described previously by our group (32). At 0.1 mg, the inactive mutant Y222F is statistically insignificant in terms of inhibiting the mucoid phenotype for all of the isolates, confirming that the catalytic activity of the enzyme is responsible for mucoid phenotype inhibition (Fig. 1B through D). Figure 1E demonstrates that Smlt1473 was additionally able to inhibit the mucoid phenotype of well-defined mucoid strain PDO300 that is an engineered variant of PAO1. Similarly to UVA 55009 and UVA 61605, Smlt1473 inhibits the mucoid phenotype of PDO300 more efficiently at 0.1 mg compared with 3 μg. As PDO300 is extremely robust at alginate production, 0.2 mg of WT Smlt1473 was also tested for its inhibitory effects. As displayed in Fig. 1E, 0.2 mg Smlt1473 behaves similarly to 0.1 mg Smlt1473 against PDO300. These data indicate an upper limit for the enzyme concentration that will inhibit alginate formation, but the specific, maximal concentration varies across isolates. Finally, Fig. 1F shows that the mucoid phenotype for UVA 84977 is neither inhibited by 3 μg nor 0.1 mg Smlt1473. However, 0.2 mg Smlt1473 shows a robust inhibitory effect against the mucoid phenotype of UVA 84977. Overall, these results show that Smlt1473 can inhibit the mucoid phenotype of *P. aeruginosa* across all clinical isolates, but the concentration necessary for maximal efficacy varies across different isolates.

### SEM images of Smlt1473-treated *P. aeruginosa*

**Fig 2:**
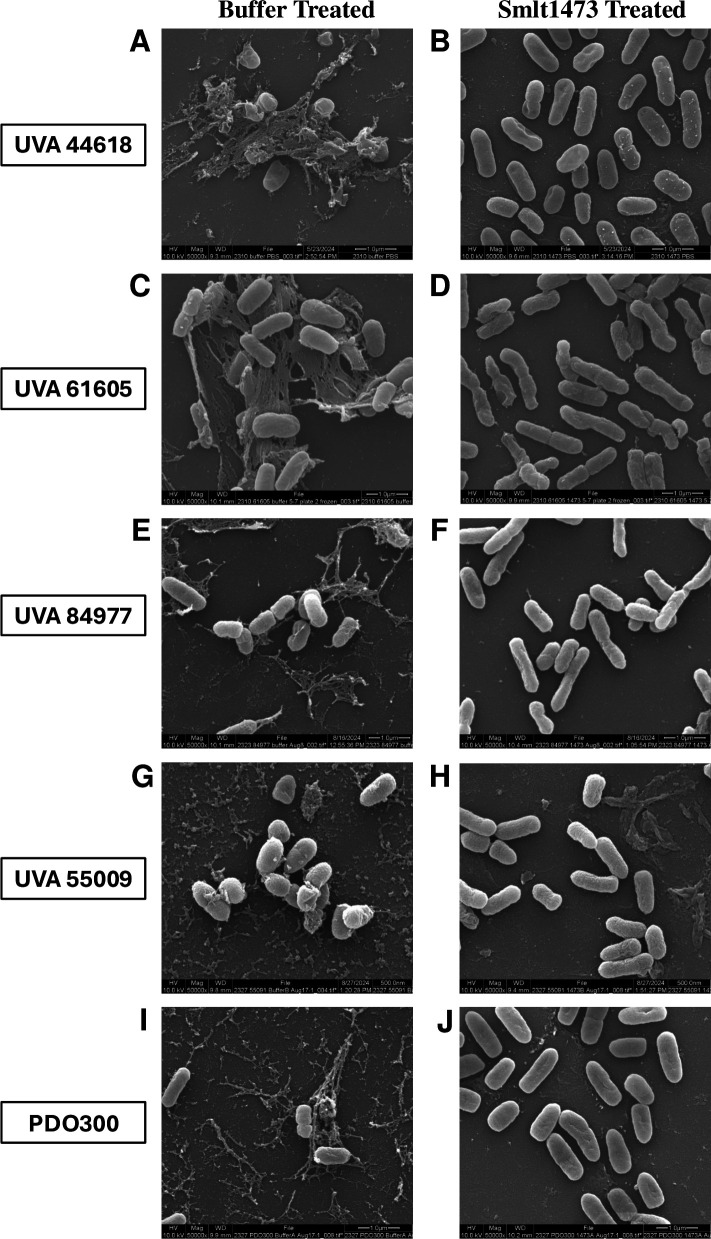
SEM images of all mucoid *P. aeruginosa* isolates treated with Smlt1473 and buffer showing enzymatic inhibition of mucoid phenotype. Samples were grown in the presence of enzyme or buffer, transferred to 12 mm glass slides, glutaraldehyde fixed, and imaged using SEM. Each set of images is shown at 50,000× magnification. The left side of the figure panel depicts samples that were grown in the presence of buffer, whereas the right side shows samples that were grown in the presence of Smlt1473. (A and B) UVA 44618, (C and D) UVA 61605, (E and F) UVA 84977, (G and H) UVA 55009, and (I and J) PDO300.

To visualize, at a more microscopic level, the effects that Smlt1473 has on *P. aeruginosa* mucoid isolates, SEM was performed on Smlt1473-treated and untreated samples. *P. aeruginosa* was grown on agar plates coated with either buffer, 0.1 mg Smlt1473, or 0.2 mg Smlt1473 and left to incubate for 24 h at 37°C. Due to results from the quantitative data, UVA 84977 and strain PDO300 were both grown in the presence of 0.2 mg Smlt1473 for SEM imaging, whereas the others were grown in the presence of 0.1 mg Smlt1473. Samples were transferred to 12 mm glass slides, glutaraldehyde fixed, and imaged using SEM. SEM was performed for each isolate and images that represent the quantitative data in Fig. 1 were chosen. It is important to note that Smlt1473 does not completely eradicate alginate secretion but on average leads to a significant decrease. Figure 2 shows SEM images for each isolate at 50,000× magnification. For UVA 44618, buffer-treated cells (Fig. 2A) are heavily intertwined and surrounded by thick EPS. However, for Smlt1473-treated cells (Fig. 2B), there is minimal EPS observed, and the cells are significantly less connected to each other. These observations hold true for all of the other isolates as well (Fig. 2C through J), and consistent with our quantitative data from Section 3.2, these images show that Smlt1473 is able to inhibit alginate secretion. Thus, SEM further supports the ability of Smlt1473 to inhibit the mucoid phenotype of *P. aeruginosa* for all of the mucoid strains tested in this study.

### Smlt1473 degrades *P. aeruginosa* alginate

**Fig 3:**
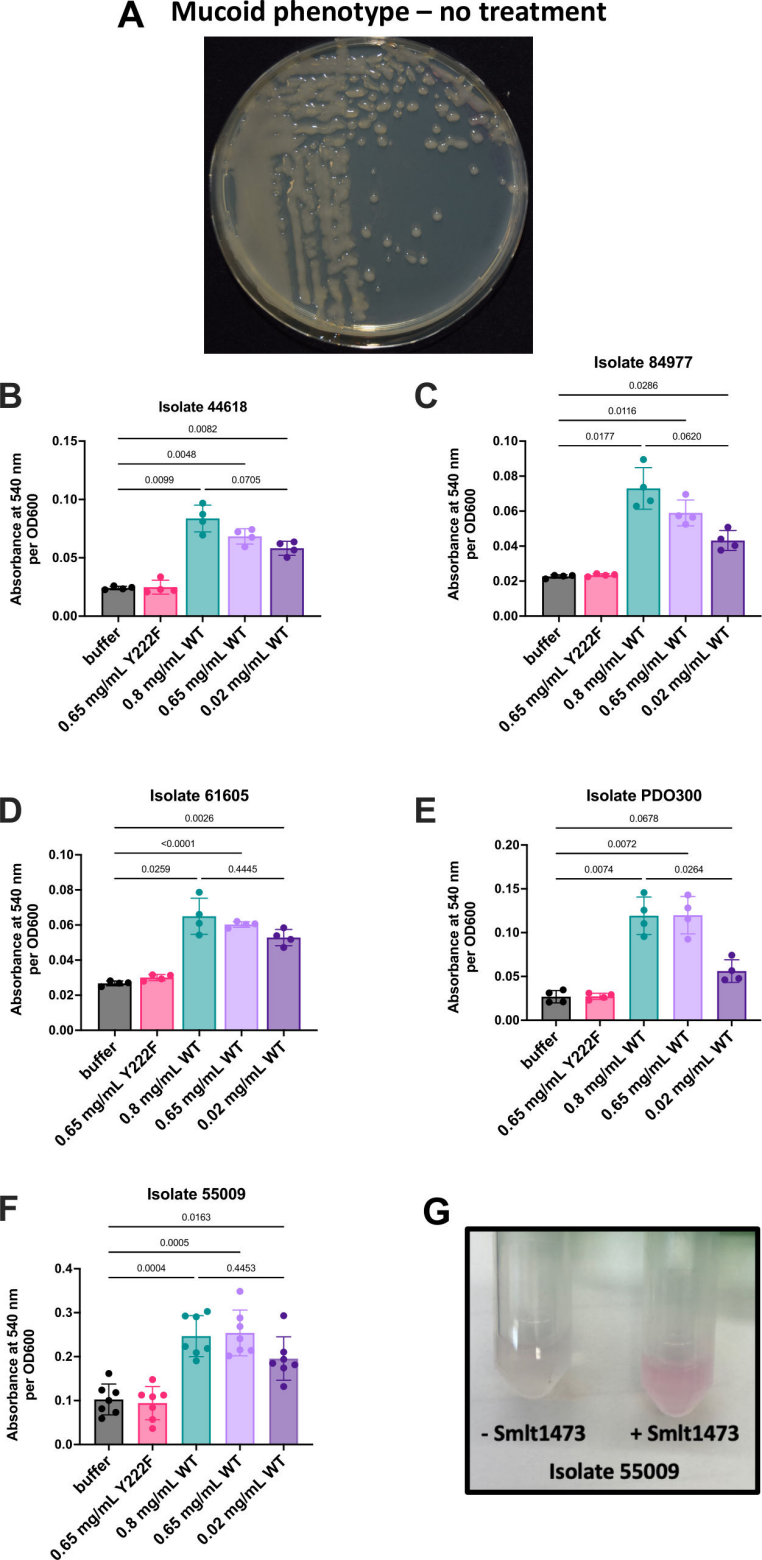
Smlt1473 degrades the mucoid biofilm of *P. aeruginosa* after it has been established on a surface. (**A**) UVA 61605 biofilm after 24 h of growth showing a prominent raised, mucoid phenotype. (**B–F**) Each *P. aeruginosa* isolate was grown on LB agar for 24 h at 37°C with no treatment to develop an established mucoid phenotype, as shown in panel A. Plate contents were collected, and the alginate-containing biofilm was used as the substrate in the TBA assay. Upon addition of enzyme, alginate is depolymerized via β-elimination mechanism where unsaturated products react with thiobarbituric acid to create a pink chromogen with absorbance at 540 nm. High A540 corresponds to greater alginate depolymerization, and low A540 corresponds to minimal alginate depolymerization. The results presented are means and standard deviations, and statistical analysis was performed using a one-way Welch’s ANOVA and Dunnett’s T3 multiple comparison post-hoc tests. (**G**) Representative image displaying the pink chromogen as a result of the addition of Smlt1473 to UVA 55009 biofilm mixture (right) compared with the addition of buffer to the mixture (left).

To examine if Smlt1473 could degrade alginate secreted by *P. aeruginosa*, we grew each clinical isolate on untreated agar plates for 24 h (Fig. 3A). Contents from the agar plates were collected and used as substrates in the TBA assay. The TBA assay is an established method to measure the formation of unsaturated products formed by alginate lyase-catalyzed depolymerization of alginate, and details of the assay are provided in Materials and Methods. As a control to prove Smlt1473 activity against alginate substrates, the TBA assay was conducted using a standard solution made from purified alginate from Sigma (Fig. S1). Similarly, this assay was used to investigate the ability of Smlt1473 to depolymerize the heterogeneous secreted fractions from various *P. aeruginosa* clinical isolates. Figure 3B shows that for isolate UVA 44618, 0.8 mg/mL, 0.65 mg/mL, and 0.02 mg/mL WT Smlt1473 all significantly degrade the alginate secreted by *P. aeruginosa* compared with the buffer control and the inactive mutant (Y222F). Similarly, for isolates UVA 84977, UVA 61605, engineered strain PDO300, and UVA 55009, all WT Smlt1473 concentrations tested were able to degrade the secreted EPS fractions (Fig. 3C through F). Figure 3C shows the colorimetric output for the TBA assay where the EPS mixture of UVA 55009 treated with sodium phosphate buffer (left) remains clear, and the EPS mixture treated with 0.8 mg/mL Smlt1473 (right) turns pink, indicating double bond formation and thus alginate depolymerization via Smlt1473 activity. For each isolate, the buffer condition and inactive mutant, Y222F, behave nearly identical, proving that, similar to the inhibition study, catalytic activity of the enzyme is responsible for the degradation effects shown. For all isolates tested, there is no significant difference in degradation activity between 0.8 mg/mL and 0.65 mg/mL Smlt1473; however, differences do exist between the highest (0.8 mg/mL) and lowest (0.02 mg/mL) concentrations. For UVA 55009 and UVA 61605, there is no significant difference in degradation activity between high and low Smlt1473 concentrations; however, there are noticeable differences for UVA 44618, UVA 84977, and engineered strain PDO300 between these concentrations. These data demonstrate that Smlt1473 can degrade established *P. aeruginosa* alginate after it has been secreted by the bacteria for all isolates tested, although the enzyme concentration needed for efficacy depends on the specific isolate.

### Characterization of EPS by carbohydrate content and alginate composition

**Fig 4:**
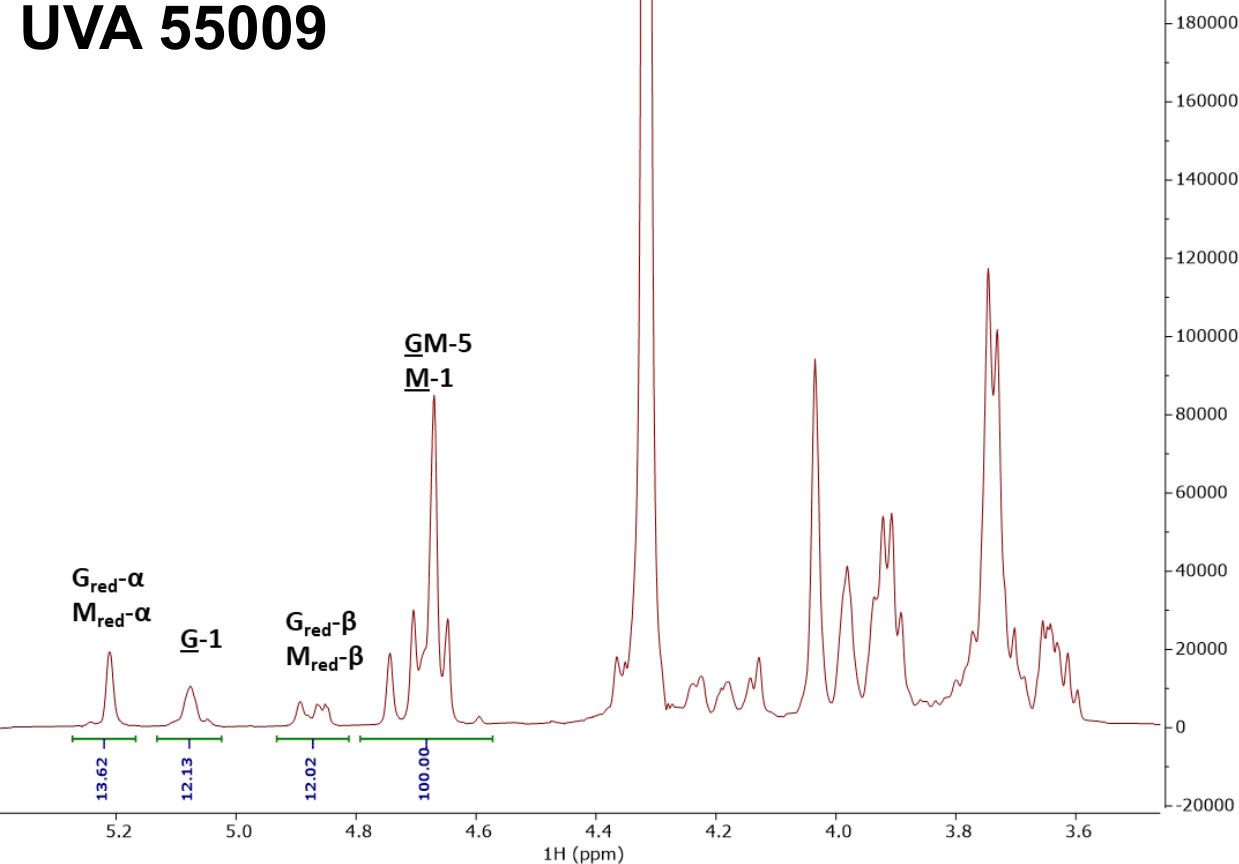
^1^H NMR spectrum of acid-hydrolyzed alginate sample from UVA 55009. Peaks in the 4.6–5.3 ppm range correspond to those of the M and G residues in the alginate sample. The integral value for the peak in the 5.02–5.13 ppm range is 12.13, which is roughly half of that for the other isolates. This is the defining peak that separates the M-to-G ratio of 55009 from the others.

**Fig 5:**
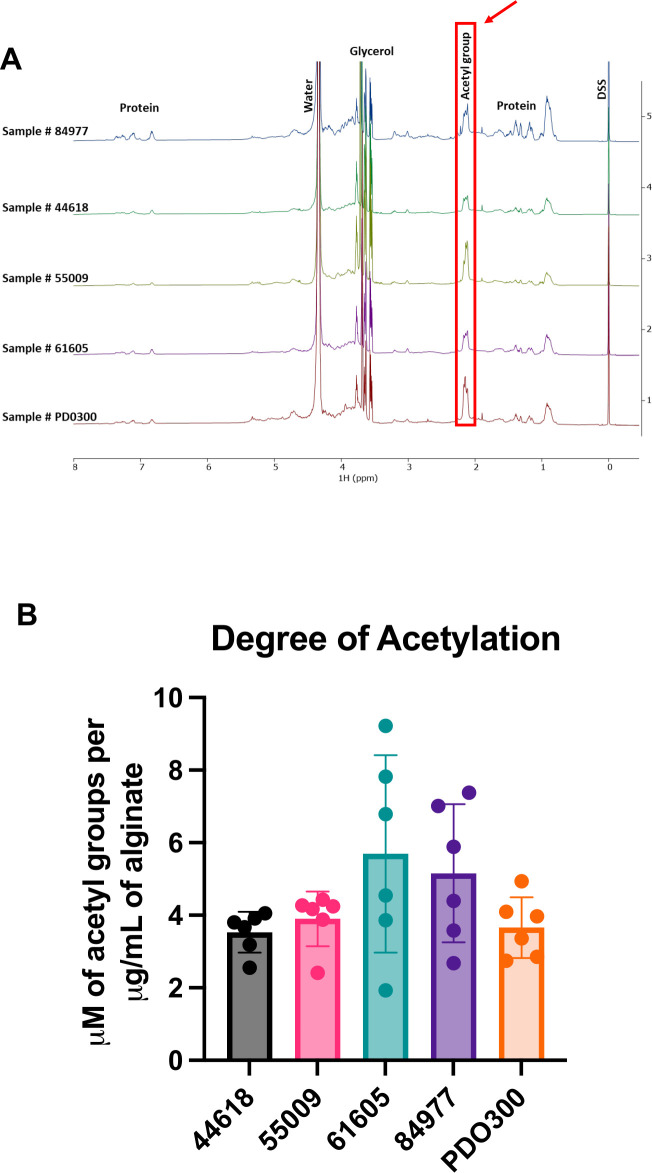
Stacked ^1^H NMR spectra of all five *P. aeruginosa* isolates showing acetylation and quantitative determination of degree of acetylation. (**A**) Peaks around 2.12ppm are a result of the acetyl group of acetylated sugars, indicating all of the isolates in this study are comprised of acetylated alginate. (**B**) The degree of acetylation was quantitatively determined using a method previously described, further proving that all biofilm samples have some fraction of acetylated alginate. Data in Fig. 2 shows that Smlt1473 degrades established mucoid biofilm, and in combination with these NMR and degree of acetylation results, we can conclude that Smlt1473 is effective against acetylated alginate.

Our prior characterization of Smlt1473 indicated a preference for depolymerization of mannuronic acid (M)-containing blocks within alginate. In our current study, Smlt1473 has variable effects on EPS formation and removal, depending on the concentration of Smlt1473 that is used against a specific isolate. For example, we show that for UVA 44618, 0.1 mg and 3 μg inhibit the mucoid phenotype almost identically (Fig. 1B). However, for the rest of the isolates tested, 0.1 mg Smlt1473 inhibits EPS production more efficiently than 3 μg Smlt1473 (Fig. 1C through F). For all isolates tested, there is no significant difference in degradation activity between 0.8 mg/mL and 0.65 mg/mL Smlt1473. However, although there is no significant difference between 0.8 mg/mL and 0.02 mg/mL Smlt1473 for UVA 61605 or UVA 55009, there is a difference in degradation activity between these concentrations for UVA 44618, UVA 84977, and engineered strain PDO300. Given these data, we were interested in determining whether changes in total alginate levels or variations in M-to-G ratio within alginate for a given clinical isolate could help explain the observed variability in Smlt1473 effects. Table 2 summarizes the total carbohydrate composition of the EPS sample isolated from each strain used in this study. The EPS of all five strains is dominantly comprised of alginate, with no significant levels of other key EPS, such as Pel or Psl, that are also known to play important roles in *P. aeruginosa* biofilm structure (33). In a study conducted by Wozniak et al., the same assay described in our manuscript was used to explore the carbohydrate monomer composition profiles of various *P. aeruginosa* strains. In this study, it is shown that the assay can indeed detect levels of glucose, rhamnose, and mannose that are attributed to Psl, as well as the presence of amino sugars attributed to Pel (34). This result proves that the assay is indeed able to detect other monosaccharides present in Psl or Pel; however, our samples just did not contain these monosaccharides. Overall, our data indicate that differences in total alginate levels do not explain the varying effects of high and low Smlt1473 concentrations for alginate inhibition and removal across the clinical isolates. Since previous work has shown Smlt1473 prefers to depolymerize mannuronic acid (M) residues of alginate, we next sought to investigate the M-to-G ratios of the alginate-dominant EPS. Four of the five strains (PDO300, UVA 44618, UVA 61605, and UVA 84977) have similar M-to-G ratios between 3.28 and 3.68 (Table 3). However, UVA 55009 is distinct in that it is significantly enriched in mannuronic acid with an MM percentage of 75% compared with the other isolates that range from an MM percentage of 53%–57%. Ultimately, this resulted in an M-to-G ratio of 7.24 for UVA 55009, more than double that of the other four strains (Table 3; Fig. 4). None of the samples contained detectable levels of consecutive G residues (GG blocks), which is typical of alginate isolated from *P. aeruginosa* (Table 3) (35). All five EPS samples exhibited strong ^1^H NMR peaks characteristic of acetylation (Fig. 5A), and the degree of acetylation was quantitatively determined using previously described methods (36, 37), confirming that all samples contained significant levels of acetylated alginate (Fig. 5B). Overall, the alginate-dominant EPS samples isolated do not show differences in total alginate levels, and only one of the five show a distinct difference in M-to-G ratio compared with the others. No significant differences in acetylation across the clinical isolates are observed either. Therefore, total alginate content, M-to-G ratio, and degree of acetylation are not major contributors to why different concentrations of Smlt1473 have varying effects on alginate inhibition and removal across the clinical isolates.

**TABLE 2:** Amount and mole percentage of each monosaccharide detected in the *P. aeruginosa* biofilm samples

	PDO300 (250 mg)		61605 (350 mg)		55009 (190 mg)		44618 (400 mg)		84977 (300 mg)	
Glycosyl reside	Mass (mg)	Mol %	Mass (mg)	Mol %	Mass (mg)	Mol %	Mass (mg)	Mol %	Mass (mg)	Mol %
Rhamnose	0.3	0.2	0.1	0.1	0.3	0.4	0.4	0.3	0.2	0.1
Fucose	n.d.^*a*^	n.d.	n.d.	n.d.	n.d.	n.d.	n.d.	n.d.	n.d.	n.d.
Xylose	0.4	0.3	0.8	0.8	0.5	0.8	0.7	0.6	0.6	0.6
Galactose	1.2	0.8	2.6	2.2	0.5	0.6	1.5	1.0	1.2	1.0
Glucose	1.8	1.2	3.3	2.8	2.0	2.3	1.4	0.9	1.8	1.5
N-acetyl glucosamine	0.3	0.2	0.4	0.4	n.d.	-	0.4	0.3	0.3	0.3
Alginate	160.5	97.3	119.3	93.7	87.6	95.9	149.5	96.9	121.1	96.5
Sum	164.6	100	126.5	100	90.0	100	153.9	100	125.1	100
Carbohydrate content in sample	59%		33%		43%		35%		38%	

^*a*^ n.d., not detectable.

**TABLE 3:** Fractional composition and M-to-G ratio for all of the alginate dominant *P. aeruginosa* biofilm samples^*a*^

	Composition		Doublet frequency			
Sample	F_G_	F_M_	F_GG_	F_MM_	F_MG_ = F_GM_	M-to-G ratio
PDO300	0.21	0.79	0	0.57	0.21	3.68
61605	0.23	0.77	0	0.54	0.23	3.35
55009	0.12	0.88	0	0.75	0.12	7.24
44618	0.23	0.77	0	0.53	0.23	3.28
84977	0.23	0.77	0	0.54	0.23	3.39

^*a*^ FG, fraction of singlet guluronic acid; Fm, fraction of singlet mannuronic acid; FGG, fraction of doublet guluronic acid; FMM, fraction of doublet mannuronic acid; M, mannuronic acid; G, guluronic acid.

## DISCUSSION

Adhesion to a surface is the first step in biofilm formation, and once this occurs, bacteria form a tightly linked network that enhances resistance mechanisms (5). Once bacteria have entered the sessile state, they become increasingly difficult to treat compared with their planktonic counterparts (38). In this study, we show that Smlt1473 can inhibit the formation of alginate, a dominant component of *P. aeruginosa* biofilm, from various clinical isolates and an engineered strain. However, inhibition data suggest that enzyme concentration plays a role in treatment efficacy depending on the strain. Biofilm formation is a challenging process to target as it involves many factors including bacterial motility, quorum-sensing, and polysaccharide synthesis (39). Our current results indicate alginate is the dominant EPS present in all tested strains of *P. aeruginosa*, suggesting it is the primary substrate for Smlt1473 in this context. Alginate degradation by Smlt1473 also helps explain the observed difference in colony morphology and measured reduction in uronic acid content when bacteria are grown in the presence of Smlt1473. Our prior work characterizing Smlt1473 demonstrated robust *in vitro* alginate degrading activity, again consistent with our current results on alginate-dominant EPS mixtures (24). In CF patients where mucoid biofilm is common and a major determinant of virulence, overproduction of alginate occurs later in biofilm development, whereas initial colonization by *P. aeruginosa* involves biofilm with a distinctly different, non-alginate EPS composition (34). Thus, it is interesting to observe that pre-treatment with Smlt1473 is able to decrease alginate secretion; this may be related in part due to variation in the rate of alginate synthesis among the strains or other secreted factors unique to individual strains able to overcome effects of Smlt1473 treatment. We do not observe any growth inhibition due to treatment with Smlt1473 (Fig. S2), which is consistent with Smlt1473 having its primary effect on alginate exopolysaccharide rather than the cells.

Since it is often difficult to inhibit *P. aeruginosa* colonization, an alternative solution is to degrade established, mucoid biofilms to facilitate removal. Our results demonstrate Smlt1473 can degrade established *P. aeruginosa* alginate, which dominates the composition of the secreted fractions from all five strains tested (Fig. 3). Results also suggest that, similar to the inhibition data, concentration efficacy may differ among strains. For example, the lowest concentration (0.02 mg/mL) behaves most similarly to the highest concentration (0.8 mg/mL) for UVA 61605 and UVA 55009, where it is not as effective as the highest concentration for the other strains. However, given that Smlt1473 has robust alginate degrading activity *in vitro* and all strains tested have EPS that is dominantly comprised of alginate (Table 2), observing degradation activity across all five isolates is consistent with expectation. To add, all five EPS samples show acetylation (Fig. 5A and B), which has been described previously in increasing the hydrophobicity of *P. aeruginosa* mucoid biofilm, enhancing clustering of cells within the biofilm, and reducing alginate lyase efficacy (40). Catalytically inactive forms of alginate lyases have been shown to disperse *P. aeruginosa* biofilm (41); however, in the current study, we demonstrate that catalytic activity is essential for the inhibition and degradation of alginate-dominant EPS (Fig. 1 and 3). Moreover, unlike other alginate lyases characterized for biofilm removal activity (23), Smlt1473 is able to degrade established alginate across all five strains, albeit with varying enzyme concentrations needed for efficacy.

Another potential reason for variation in Smlt1473 activity for both EPS inhibition and degradation is variation in alginate composition. Alginate is comprised of 1,4-linked β-D-mannuronic (M) and α-L-guluronic (G) acids, and the final polysaccharide can be arranged in M-blocks (MM), G-blocks (GG), or MG-blocks (MG or GM) (42). In patients with CF, alginate is enriched in M, which makes alginate more elastic (42). This increase in mucoid biofilm elasticity due to greater amounts of M is also thought to confer advantages in terms of persistence in the lung (12). We previously determined that Smlt1473 has robust lyase activity against alginate, with a preference for poly-M rather than poly-G (24). Interestingly, when we measured the M-to-G ratio of alginate isolated from each of the five mucoid samples, all of them were enriched in M versus G, but four of the five samples had essentially identical M-to-G ratios (Table 3). However, strain UVA 55009 had a significantly higher M-to-G ratio and enrichment of M-blocks (Table 3; Fig. 4). Of note, the lowest concentration of Smlt1473 (0.02 mg/mL) behaves similarly to the highest (0.8 mg/mL) for strain UVA 55009 with the highest M-to-G ratio, consistent with the greater poly-M-degrading activity for Smlt1473. However, this trend is also observed for UVA 61605, which has an M-to-G ratio similar to that of the other clinical isolates. Moreover, in terms of alginate inhibition, strain UVA 44618 and engineered strain PDO300 had greater inhibition at lower Smlt1473 concentration than strain UVA 55009; however, UVA 44618 and PDO300 have lower M-to-G ratios than strain UVA 55009, suggesting that although M-to-G ratio may play a role in Smlt1473 degradation of alginate, other factors such as rate of alginate production or percentage of non-EPS components may be important.

Aside from acetylation (Fig. 5), there may also be differences in other EPS characteristics in the early stages of biofilm formation that play an important role in biofilm architecture and accessibility of alginate to degradation. Psl and Pel are two polysaccharides that are often studied in biofilms of non-mucoid strains, but prior work indicates they also can be present in mucoid biofilm (43, 44). We did not detect the presence of saccharides associated with Psl or Pel in mucoid biofilm isolates (Table 2) but cannot rule out the possibility of low levels in mucoid strains or their presence at higher levels in our inhibition studies before the mature biofilm is formed. Future studies determining biofilm polysaccharide composition during the transition to mucoid state, as well as changes in M-to-G ratio during this transition, may provide further insight into how Smlt1473 is able to inhibit and degrade *P. aeruginosa* biofilms. Additionally, adaptations that clinical isolates make to overcome treatment with Smlt1473, the rate at which alginate is secreted, and alginate polysaccharide sequence/chain length for each isolate may provide further insight into the characteristics/environment needed for optimal Smlt1473 activity against specific *P. aeruginosa* alginate. Although alginate is shown to be a dominant component of all of the isolates in this study, variations in percentages of non-EPS components such as DNA and protein could also contribute to differences in enzyme efficacy.

In conclusion, we demonstrate that the polysaccharide lyase, Smlt1473, can inhibit alginate formation in all *P. aeruginosa* isolates tested but that the concentration able to do so is strain-dependent. Additionally, we demonstrated Smlt1473 can degrade mature and established alginate mixtures for all five isolates, but again, the concentration efficacy varies across isolates. We used the carbazole assay (43, 45) for inhibition experiments to quantitatively determine uronic acid, that is, alginate, concentration as a result of pre-treatment with Smlt1473 and the TBA assay (32, 46) for degradation experiments, which gives a direct, quantitative readout of product concentration from polysaccharide lyase-catalyzed alginate depolymerization rather than indirect staining methods such as the crystal violet assay (47–49). Although carbohydrate composition, alginate M-to-G ratio, and acetylation were explored, additional studies are needed to determine the causative agents that can predict Smlt1473 efficacy. Future studies will need to further explore characteristics of mucoid biofilm that can better predict enzyme efficacy, including rate of alginate secretion, alginate sequence, and chain length. Additionally, differences in non-EPS components such as DNA and protein among the isolates could be explored to see if there is a link to enzyme efficacy based on these factors. Acquired mutations in genes responsible for the mucoid phenotype such as mucA or mucB, as well as differences in genetic background across the clinical isolates, could also be explored in a future study for insight into enzyme efficacy.

It is important to emphasize that this study used a range of *P. aeruginosa* clinical isolates from the UVA Health System (Table 1) to demonstrate Smlt1473 robustness, as opposed to other studies which commonly use PA14, PAO1, or FRD1 strains (23, 41, 50–52). Additionally, although Smlt1473 can be characterized as an alginate lyase, it is unique in that it can degrade other polysaccharides as well (24). This characteristic differentiates Smlt1473 from other alginate lyases in that Smlt1473 can be used more broadly against polysaccharides from a variety of pathogens rather than being constrained to alginate producers such as *P. aeruginosa*. We have also shown that Smlt1473 is active against acetylated alginate, which differs from some lyases, including A1-II from S*phingomonas sp*. and AlgL from *P. aeruginosa* that prefer non-acetylated alginate (23). This characteristic is important for treating *P. aeruginosa* as it secretes O-acetylated alginate during chronic lung infection (53). A study conducted by Blanco-Cabra et al. claims that broad spectrum (polyM/G) enzymes, such as Alg2A from *Flavobacterium sp*. S20 and A1-II’ from *Sphingomonas sp*. A1, are the most active (23). However, our work shows that Smlt1473, which preferentially degrades polyM, is highly efficacious against five mucoid isolates of *P. aeruginosa*. This result highlights that high activity toward both M and G is not always required for efficacy against alginate produced by *P. aeruginosa*. To add, work done by Blanco-Cabra et al. comprehensively looks at alginate lyases and compares five different enzymes; however, the study does not explore if the lyases are toxic to human cells (23). We have shown that our enzyme does not affect the viability of bronchial epithelial cells by testing on the BEAS-2B cell line (Fig. S4), which is an important metric for possible future treatment regimens using Smlt1473. Overall, our work demonstrates successful inhibition and degradation of *P. aeruginosa* alginate by polysaccharide lyase Smlt1473 over a wide range of clinical isolates, which may have utility in removing mucoid biofilm associated with drug-resistant infections. We recognize the opportunity to add to this work and hope to do so in the future by exploring the synergism of Smlt1473 treatment with different classes of antibiotics to enhance the utility of the enzyme and further contribute to the field.

## MATERIALS AND METHODS

### Molecular biology

An *E. coli* codon-optimized nucleotide sequence of Smlt1473 (GenBank accession number CAQ45011) was synthesized and cloned into pET-28a(+) using NcoI/XhoI restriction sites (GenScript). The coding sequence contains N- and C- terminal hexahistidine tags suitable for our protein purification method via affinity chromatography. The plasmid was transformed into *E. coli* DH5a for DNA isolation and *E. coli* BL21 for protein expression. For site-directed mutagenesis, primers were designed using PrimerX, and amino acid substitutions were made using QuikChange II site-directed mutagenesis kit (Agilent). All sequences were verified by DNA sequencing (Eurofins).

### Protein expression and purification

Smlt1473 cloned into pET-28a(+) was transformed into *E. coli* BL21 cells. Cells were grown in 10 mL of LB with kanamycin (50 μg/mL) overnight at 37°C and shaking at 200 rpm. Cells were harvested by centrifugation at 3,000 *g* for 10 min and then resuspended in 1 mL of fresh LB media. One milliliter of the cell suspension was used to inoculate a flask with 250 mL of LB. Flasks were incubated at 37°C shaking until an OD_600_ between 0.6 and 0.8 was reached. Protein expression was induced using 1 mM isopropyl β-D-1-thiogalactopyranoside (IPTG), and then, flasks were incubated at 20°C shaking for 16–18 h. Cells were harvested by centrifugation at 5,000 *g* for 15 min, the supernatant was discarded, and the cells were resuspended in lysis buffer (50 mM Tris-HCl, 100 mM NaCl, 5% vol/vol glycerol) prior to sonication. Thirty milliliters of lysis buffer were used for 500 mL of expression culture. Sonication was performed with 20 s pulses for 20 min. After sonication, the mixture was clarified by centrifugation at 10,000 × *g* for 15 min. The supernatant containing protein was collected, and the insoluble fraction was discarded. The supernatant containing protein was purified via immobilized-metal ion affinity chromatography (IMAC). The column was equilibrated with five column volumes of 10 mM imidazole/IMAC solution before adding the 0.2 μm filtered protein-containing supernatant. The protein was separated using imidazole elutions of 10, 20, 30, 50, 100, and 250 mM; 100 mM typically yielded the highest concentration of Smlt1473. Each fraction was analyzed by SDS-PAGE with Precision Plus protein all-blue molecular weight standard (Bio-Rad) used as a molecular weight standard. The fractions with the highest purity were then dialyzed in 3.5 L of 20 mM sodium phosphate buffer (pH 8) overnight at 4°C with stirring. Protein concentration was determined by using either the bicinchoninic acid (BCA) protein assay or by measuring the absorbance at 280 nm and determining concentration using a calculated extinction coefficient of 74,495 M^−1^ cm^−1^. Protein aliquots were stored at −20°C to be used in future experiments.

### Enzyme activity assay

To ensure Smlt1473 was functional post-purification, the thiobarbituric acid (TBA) assay, with some adaptations, was used (46). Alginate lyases depolymerize alginate via a β-elimination mechanism, which results in the formation of a double bond, that is, an unsaturated product that ultimately reacts with thiobarbituric acid, leading to a pink chromogen with absorbance at 540 nm. Thus, absorbance at 540 nm correlates to alginate depolymerization (32, 46).

Briefly, the assay used a 2 mg/mL alginate solution created from purified alginic acid (Sigma), 0.025 M periodic acid in 0.125 N sulfuric acid, 2% arsenite in 0.5 N HCl, and 0.45 µm filtered 0.71% 2-thiobarbituric acid in 0.7 mM NaOH. Due to light sensitivity, 0.71% TBA solution was stored in a dark container. Fifty microliters of 2 mg/mL alginate were added to a 1.5 mL microcentrifuge tube along with 40 μL of the enzyme, mixed, and left to incubate for 30 min at room temperature. After incubation, 15 μL of 0.025 M periodic acid was added and mixed, and the tubes were incubated for an additional 40 min at room temperature. To terminate the reaction, 30 μL of 2% sodium arsenite was added, mixed, and left to incubate for 2 min. For activity detection, 290 μL of 0.71% TBA reagent was added to each tube and placed in a heat block at 90°C for 10 min. Following incubation, tubes were centrifuged for 2 min, and then, 150 μL was added into a 96-well plate to measure absorbance at 540 nm.

### Bacterial strains

Five mucoid isolates of *P. aeruginosa* were used in this study. The clinical isolates used in this study (UVA 44618, UVA 61605, UVA 55009, and UVA 84977) are from a collection of isolates from the University of Virginia hospital acquired from February 2019 to February 2020, as described in a publication by Dunphy et al. in 2021 (54). In addition to what was included in Table 1, the publication reports bacterial susceptibility profiles, virulence-linked morphological phenotypes, as well as patient metadata including antimicrobial prescription history. Several of these isolates were also sequenced and deeply characterized with computational methods in a recent work published by Islam et al. in 2024 (55). Susceptibility to antibiotics as well as determination of the virulence-linked morphological phenotypes were performed by the Clinical Microbiology Laboratory at UVA. The fifth strain, PDO300, was kindly provided by Dr. Joanna Goldberg at Emory University School of Medicine. PDO300 is a defined isogenic derivative of PAO1 that was engineered to have the mucA22 allele, allowing it to constitutively overproduce the alginate exopolysaccharide (56). From the whole genome sequencing data, there appear to be differences in mucA and mucB between UVA 44618, UVA 61605, and UVA 55009. These genes are known to be important for the conversion to the mucoid phenotype; hence, differences in these genes could account for the variability in alginate production among the clinical isolates (57). There are no observed differences in mucC or mucD between the clinical isolates that were sequenced.

### Mucoid phenotype inhibition assays

Blood agar or LB agar plates were coated with 20 mM sodium phosphate buffer, 0.2 mg wild-type (WT) Smlt1473, 0.1 mg WT Smlt1473, 3 μg WT Smlt1473, or 0.1 mg inactive mutant Y222F using glass spreaders to distribute the treatment across the entire plate. Plates were dried, and *P. aeruginosa* was streaked using an inoculating loop either from liquid culture or frozen stock, depending on the isolate. For liquid culture, 5 mL of LB was inoculated using frozen stock and grown at 37°C for 18 h at 150 rpm. Alternatively, an inoculating loop was used to scrape *P. aeruginosa* directly from frozen stock and plated. Agar plates were wrapped in parafilm to avoid desiccation and incubated at 37°C for 24 h. Following incubation, all plate contents were collected using 10 mL of 0.85% NaCl and vortexed to ensure a homogenous solution before use in the carbazole assay to quantify total uronic acid content (58). OD_600_ of the suspension was measured undiluted, 5-fold diluted, 10-fold diluted, and averaged for normalization purposes. Dilutions were used where undiluted sample OD_600_ >0.8.

Uronic acid content was quantified using the carbazole assay based on an established protocol to quantify the amount of alginate in each sample in the presence and absence of Smlt1473 pre-treatment (58). Alginate is made up of two different uronic acids—mannuronic and guluronic acid. The carbazole assay (used in this study) is a common method to detect uronic acid, which in this case corresponds to alginate content (58–61). The sample solution is added to ACS-grade sulfuric acid, which hydrolyzes the polysaccharides in the mixture to their uronic acid monomer components. Carbazole is then added which reacts with the uronic acid monomers to form a pink/purple solution that is measured by absorbance at 530 nm, indicating the presence or absence of uronic acid (in our case, alginate). It was reported by Wozniak et al. that alginate is not a significant component of the EPS matrix for PA14 (34). Therefore, to prove specificity to uronic acid, we conducted the carbazole assay with supernatant from nonmucoid *P. aeruginosa* strain PA14 to show that when uronic acids are not present, detection by carbazole assay is very minimal (Fig. S3). For the assay, 5 mL of 2 M BO_3_^−3^ was made by dissolving 24.74 g H_3_BO_3_ in 45 mL of 4 M KOH with heat, and then deionized water was added to a total volume of 200 mL. Next, the 2 M BO_3_^−3^ solution was added to ACS grade H_2_SO_4_ to create a 95% H_2_SO_4_ solution; H_2_SO_4_ must be ACS grade for the reaction to occur. Finally, 0.1% carbazole (Alfa Aesar) reagent was made. Alginate standards were created using purified alginic acid (Sigma) diluted in 0.85% NaCl to concentrations of 100–1,300 μg/mL depending on the amount of alginate produced. For reactions, 3 mL of H_2_SO_4_ was added to glass tubes on ice, followed by 350 μL of alginate standard or mucoid suspension collected from agar plates. The tubes were vortexed to mix, and then, 100 μL of 0.1% carbazole was added. The tubes were again vortexed and then incubated in a heat block at 55°C for 30 min for reaction product to occur. After incubation, the tubes were vortexed, and 200 μL was transferred to a 96-well plate for absorbance measurements at 530 nm. For analysis, values at 530 nm were used from an averaged standard curve to determine uronic acid concentration, that is, alginate for each isolate and treatment. To account for differences in growth, the results are reported as uronic acid concentration divided by OD_600_.

### Electron microscopy

*P. aeruginosa* was grown on agar plates to support mucoid phenotype formation. Agar plates were coated with either buffer as a control or 0.1 mg Smlt1473 (similar to in Section 2.5). Polycarbonate membrane filters were placed on the agar plate prior to streaking the bacteria for ease of transfer to glass slides for imaging, and plates were left to incubate for 24 h at 37°C. Prior to bacterial adherence, slides were pre-treated with 1 mL 0.1 mg/mL poly-D-lysine and left to incubate for 1 h. Following incubation, poly-D-lysine was aspirated, and the slides were washed with PBS and left to dry for 1 h. For bacterial transfer from agar to slides, 12 mm circle No. two slides (VWR) were placed on top of the polycarbonate membranes with bacteria and allowed to adhere for 30 min. Slides were then transferred to a 12-well plate and left to dry overnight. Two milliliters of 2.5% glutaraldehyde were slowly added and left on the slides for 1–2 h, followed by a PBS wash. The slides were then stored in 1 mL PBS at 4°C before being sent to the United States Department of Agriculture (USDA) for scanning electron microscopy (SEM) imaging. The remainder of the preparation for SEM was done at the USDA using previously described methods (62). All SEM images were collected using a Quanta 200 FEG scanning electron microscope.

### Secreted alginate degradation assay

*P. aeruginosa* isolates were streaked on LB agar from frozen stock and incubated at 37°C for 24 h. Contents of the agar plate were collected using 10 mL of 0.85% NaCl and vortexed thoroughly to ensure a homogenous solution. OD_600_ was measured undiluted, 5-fold diluted, 10-fold diluted, and averaged for normalization purposes. Dilutions were used where undiluted sample OD_600_ >0.8. To examine the degradation capability of Smlt1473 against alginate secreted by *P. aeruginosa*, the TBA assay described in Section 2.3 was used. Plate contents were collected in 3.3 mL of 0.85% NaCl (3× concentrated) so that less sample and more enzyme could be used while maintaining the same sample mass. Then, 16.7 μL of sample and 73.2 μL of buffer or treatment were used for final concentrations of 0.8 mg/mL WT Smlt1473, 0.65 mg/mL WT Smlt1473, 0.02 mg/mLWT Smlt1473, and 0.65 mg/mL Y222F. Since secreted fractions containing alginate from clinical isolates were used rather than purified alginic acid, we divided absorbance at 540 nm by OD_600_ of the initial mucoid suspension to account for differences in bacterial growth and alginate production between the trials.

As a control, purified alginic acid (Sigma) was used in the TBA assay as well to prove Smlt1473 activity against alginate substrates (Fig. S1). In total, 73.2 μL of buffer or enzyme at various concentrations was added to 16.7 μL of 2 mg/mL alginate solution, corresponding to a final concentration of 370 μg/mL, which is in the range of alginate production for the mucoid *P. aeruginosa* isolates. The rest of the assay was conducted as described in Section 2.3.

### Total carbohydrate analysis and determination of alginate composition

First, polysaccharide fractions were isolated from each *P. aeruginosa* strain. *P. aeruginosa* was grown at 37°C for 24 h on LB agar, and then, plate contents were collected with 0.85% NaCl. The mixture was centrifuged at 8,300 *g* and 22°C for 15 min to remove the cells. The supernatant was collected and incubated with 75% vol/vol ethanol at 4°C for 1 h. The formed precipitate was washed 3 times with 100% ethanol and then resuspended in 2 mL of buffer (1 mM CaCl_2_ and 2 mM MgCl_2_ in 50 mM Tris pH7.5). In total, 250 μL of DNase I and 250 μL of RNase A were added to the suspension, which then was incubated for 2 h at 37°C. Additionally, 250 μL of Proteinase K was added, and the suspension was incubated at 37°C overnight. Finally, the suspension was dialyzed against H_2_O, lyophilized, and sent to the Complex Carbohydrate Research Center (CCRC) at the University of Georgia for analysis.

Glycosyl composition analysis was performed by the CCRC using combined gas chromatography-mass spectrometry (GC-MS) of the per-O-trimethylsilyl (TMS) derivatives of the monosaccharide methyl glycosides produced from the sample by acid methanolysis using a method previously described (63). Prior to GC-MS of the TMS derivatives, samples were depolymerized and N-acetylated. Additionally, CCRC performed NMR spectroscopy for this study. Prior to spectroscopy analysis, pronase digestion and deacetylation were performed on each of the samples to remove protein contamination and acetyl groups. NMR data were acquired using standard procedures.

### Degree of acetylation

*P. aeruginosa* isolates were streaked on LB agar from frozen stock and incubated at 37°C for 24 h. Contents of the agar plate were collected using 10 mL of 0.85% NaCl and vortexed thoroughly to ensure a homogenous solution. The carbazole assay to determine alginate concentration was conducted as described in Section 2.5 as the concentration of acetyl groups would be normalized by the total alginate content.

Alginate acetylation was measured using a method previously described by Hestrin (36, 37). In total, 500 μL of mucoid sample was added to 500 μL of alkaline hydroxylamine (0.35 M NH_2_OH, 0.75 M NaOH) and incubated for 10 min. at 25°C. Next, 500 μL of 1 M perchloric acid was added, and then, 500 μL of 70 mM perchlorate in 0.5 M perchloric acid was added. Absorbance was then measured at 500 nm. A standard curve was generated using ethyl acetate where 10 mM acetyls = 192 μL of ethyl acetate in 100 mL of H_2_O (64). The concentration of acetyl groups was determined using the ethyl acetate standard curve and then divided by the concentration of alginate present in the sample to determine the degree of acetylation.

### *P. aeruginosa* growth curves

For each *P. aeruginosa* isolate, 5 mL of LB was inoculated using frozen stock and grown at 37°C for 20 h shaking at 150 rpm. Cultures were diluted to a starting OD_600_ of 0.01. Two conditions were evaluated: Smlt1473 treated and buffer treated. Seventy-five microliters of Smlt1473 or buffer was added to 125 μL of diluted culture for a final concentration of 0.47 mg/mL Smlt1473 in a total volume of 200 μL. This was the highest achievable concentration of Smlt1473 in liquid culture. Growth was monitored at 37°C for 17.5 h using the Tecan Infinite 200 PRO.

### Statistical analysis

Statistical analyses were performed using RStudio 2021.09.2 + 382 and GraphPad Prism Version 9.3.1. Values in all graphs are expressed as mean with standard deviation (SD). Comparisons were done using one-way Welch’s ANOVA and Dunnett’s T3 multiple comparison post-hoc tests due to unequal variance and sample size, respectively. The number of trials for each isolate with its corresponding experiment was dependent upon variance in mucoid phenotype production, as this varied among the clinical isolates. *P*-values are provided on the respective data figures in the manuscript.

## SUPPLEMENTAL MATERIAL

Supplemental figures aem.01853-24-s0001.pdf Figures S1 to S4.

ASM does not own the copyrights to Supplemental Material that may be linked to, or accessed through, an article. The authors have granted ASM a non-exclusive, world-wide license to publish the Supplemental Material files. Please contact the corresponding author directly for reuse.

## References

[1] Muhammad MH, Idris AL, Fan X, Guo Y, Yu Y, Jin X, Qiu J, Guan X, Huang T. 2020. Beyond risk: bacterial biofilms and their regulating approaches. Front Microbiol 11:928. doi:10.3389/fmicb.2020.00928 32508772 PMC7253578

[2] Okuda K-I, Nagahori R, Yamada S, Sugimoto S, Sato C, Sato M, Iwase T, Hashimoto K, Mizunoe Y. 2018. The composition and structure of biofilms developed by Propionibacterium acnes isolated from cardiac pacemaker devices. Front Microbiol 9:182. doi:10.3389/fmicb.2018.00182 29491850 PMC5817082

[3] Bjarnsholt T, Alhede M, Alhede M, Eickhardt-Sørensen SR, Moser C, Kühl M, Jensen PØ, Høiby N. 2013. The in vivo biofilm. Trends Microbiol 21:466–474. doi:10.1016/j.tim.2013.06.002 23827084

[4] Toyofuku M, Inaba T, Kiyokawa T, Obana N, Yawata Y, Nomura N. 2016. Environmental factors that shape biofilm formation. Biosci Biotechnol Biochem 80:7–12. doi:10.1080/09168451.2015.1058701 26103134

[5] Thi MTT, Wibowo D, Rehm BHA. 2020. Pseudomonas aeruginosa biofilms. Int J Mol Sci 21:8671. doi:10.3390/ijms21228671 33212950 PMC7698413

[6] González JF, Hahn MM, Gunn JS. 2018. Chronic biofilm-based infections: skewing of the immune response. Pathog Dis 76:fty023. doi:10.1093/femspd/fty023 29718176 PMC6251518

[7] Bridier A, Briandet R, Thomas V, Dubois-Brissonnet F. 2011. Resistance of bacterial biofilms to disinfectants: a review. Biofouling 27:1017–1032. doi:10.1080/08927014.2011.626899 22011093

[8] Pezzoni M, Pizarro RA, Costa CS. 2014. Protective role of extracellular catalase (KatA) against UVA radiation in Pseudomonas aeruginosa biofilms. J Photochem Photobiol B 131:53–64. doi:10.1016/j.jphotobiol.2014.01.005 24491420

[9] Maurice NM, Bedi B, Sadikot RT. 2018. Pseudomonas aeruginosa biofilms: host response and clinical implications in lung infections. Am J Respir Cell Mol Biol 58:428–439. doi:10.1165/rcmb.2017-0321TR 29372812 PMC5894500

[10] Malhotra S, Limoli DH, English AE, Parsek MR, Wozniak DJ. 2018. Mixed communities of mucoid and nonmucoid Pseudomonas aeruginosa exhibit enhanced resistance to host antimicrobials. mBio 9:e00275-18. doi:10.1128/mBio.00275-18 29588399 PMC5874919

[11] Surette MG. 2014. The cystic fibrosis lung microbiome. Ann Am Thorac Soc 11:S61–S65. doi:10.1513/AnnalsATS.201306-159MG 24437409

[12] Guillaume O, Butnarasu C, Visentin S, Reimhult E. 2022. Interplay between biofilm microenvironment and pathogenicity of Pseudomonas aeruginosa in cystic fibrosis lung chronic infection. Biofilm 4:100089. doi:10.1016/j.bioflm.2022.100089 36324525 PMC9618985

[13] Malektaj H, Drozdov AD, deClaville Christiansen J. 2023. Mechanical properties of alginate hydrogels cross-linked with multivalent cations. Polymers (Basel) 15:3012. doi:10.3390/polym15143012 37514402 PMC10386690

[14] Mohammed C, Lalgee L, Kistow M, Jalsa N, Ward K. 2022. On the binding affinity and thermodynamics of sodium alginate-heavy metal ion interactions for efficient adsorption. Carbohydr Polym Technol Appl 3:100203. doi:10.1016/j.carpta.2022.100203

[15] Braccini I, Grasso RP, Pérez S. 1999. Conformational and configurational features of acidic polysaccharides and their interactions with calcium ions: a molecular modeling investigation. Carbohydr Res 317:119–130. doi:10.1016/s0008-6215(99)00062-2 10498439

[16] Heriot M, Nottelet B, Garric X, D’Este M, Richards GR, Moriarty FT, Eglin D, Guillaume O. 2019. Interaction of gentamicin sulfate with alginate and consequences on the physico-chemical properties of alginate-containing biofilms. Int J Biol Macromol 121:390–397. doi:10.1016/j.ijbiomac.2018.10.025 30304700

[17] Pier GB, Coleman F, Grout M, Franklin M, Ohman DE. 2001. Role of alginate O acetylation in resistance of mucoid Pseudomonas aeruginosa to opsonic phagocytosis. Infect Immun 69:1895–1901. doi:10.1128/IAI.69.3.1895-1901.2001 11179370 PMC98099

[18] Tielen P, Strathmann M, Jaeger K-E, Flemming H-C, Wingender J. 2005. Alginate acetylation influences initial surface colonization by mucoid Pseudomonas aeruginosa. Microbiol Res 160:165–176. doi:10.1016/j.micres.2004.11.003 15881834

[19] Thallinger B, Prasetyo EN, Nyanhongo GS, Guebitz GM. 2013. Antimicrobial enzymes: an emerging strategy to fight microbes and microbial biofilms. Biotechnol J 8:97–109. doi:10.1002/biot.201200313 23281326

[20] Ramakrishnan R, Singh AK, Singh S, Chakravortty D, Das D. 2022. Enzymatic dispersion of biofilms: an emerging biocatalytic avenue to combat biofilm-mediated microbial infections. J Biol Chem 298:102352. doi:10.1016/j.jbc.2022.102352 35940306 PMC9478923

[21] Stiefel P, Mauerhofer S, Schneider J, Maniura-Weber K, Rosenberg U, Ren Q. 2016. Enzymes enhance biofilm removal efficiency of cleaners. Antimicrob Agents Chemother 60:3647–3652. doi:10.1128/AAC.00400-16 27044552 PMC4879406

[22] Albrecht MT, Schiller NL. 2005. Alginate Lyase (AlgL) activity is required for alginate biosynthesis in Pseudomonas aeruginosa. J Bacteriol 187:3869–3872. doi:10.1128/JB.187.11.3869-3872.2005 15901714 PMC1112040

[23] Blanco-Cabra N, Paetzold B, Ferrar T, Mazzolini R, Torrents E, Serrano L, LLuch-Senar M. 2020. Characterization of different alginate lyases for dissolving Pseudomonas aeruginosa biofilms. Sci Rep 10:9390. doi:10.1038/s41598-020-66293-2 32523130 PMC7287115

[24] MacDonald LC, Berger BW. 2014. A polysaccharide lyase from Stenotrophomonas maltophilia with a unique, pH-regulated substrate specificity. J Biol Chem 289:312–325. doi:10.1074/jbc.M113.489195 24257754 PMC3879554

[25] Drula E, Garron M-L, Dogan S, Lombard V, Henrissat B, Terrapon N. 2022. The carbohydrate-active enzyme database: functions and literature. Nucleic Acids Res 50:D571–D577. doi:10.1093/nar/gkab1045 34850161 PMC8728194

[26] Zhou L, Meng Q, Zhang R, Jiang B, Liu X, Chen J, Zhang T. 2022. Characterization of a novel polysaccharide lyase family 5 alginate lyase with PolyM substrate specificity. Foods 11:3527. doi:10.3390/foods11213527 36360141 PMC9655155

[27] Miyake O, Ochiai A, Hashimoto W, Murata K. 2004. Origin and diversity of alginate lyases of families PL-5 and -7 in Sphingomonas sp. strain A1. J Bacteriol 186:2891–2896. doi:10.1128/JB.186.9.2891-2896.2004 15090531 PMC387801

[28] Mahajan S, Sunsunwal S, Gautam V, Singh M, Ramya TNC. 2021. Biofilm inhibitory effect of alginate lyases on mucoid P. aeruginosa from a cystic fibrosis patient. Biochem Biophys Rep 26:101028. doi:10.1016/j.bbrep.2021.101028 34095554 PMC8165544

[29] Daboor SM, Rohde JR, Cheng Z. 2021. Disruption of the extracellular polymeric network of Pseudomonas aeruginosa biofilms by alginate lyase enhances pathogen eradication by antibiotics. J Cyst Fibros 20:264–270. doi:10.1016/j.jcf.2020.04.006 32482592

[30] Pandey S, Mahanta P, Berger BW, Acharya R. 2021. Structural insights into the mechanism of pH-selective substrate specificity of the polysaccharide lyase Smlt1473. J Biol Chem 297:101014. doi:10.1016/j.jbc.2021.101014 34358563 PMC8511899

[31] Mathee K, Ciofu O, Sternberg C, Lindum PW, Campbell JIA, Jensen P, Johnsen AH, Givskov M, Ohman DE, Søren M, Høiby N, Kharazmi A. 1999. Mucoid conversion of Pseudornonas aeruginosa by hydrogen peroxide: a mechanism for virulence activation in the cystic fibrosis lung. Microbiology (Reading) 145:1349–1357. doi:10.1099/13500872-145-6-1349 10411261

[32] MacDonald LC, Berger BW. 2014. Insight into the role of substrate-binding residues in conferring substrate specificity for the multifunctional polysaccharide lyase Smlt1473. J Biol Chem 289:18022–18032. doi:10.1074/jbc.M114.571299 24808176 PMC4140301

[33] Colvin KM, Irie Y, Tart CS, Urbano R, Whitney JC, Ryder C, Howell PL, Wozniak DJ, Parsek MR. 2012. The Pel and Psl polysaccharides provide Pseudomonas aeruginosa structural redundancy within the biofilm matrix. Environ Microbiol 14:1913–1928. doi:10.1111/j.1462-2920.2011.02657.x 22176658 PMC3840794

[34] Wozniak DJ, Wyckoff TJO, Starkey M, Keyser R, Azadi P, O’Toole GA, Parsek MR. 2003. Alginate is not a significant component of the extracellular polysaccharide matrix of PA14 and PAO1 Pseudomonas aeruginosa biofilms. Proc Natl Acad Sci U S A 100:7907–7912. doi:10.1073/pnas.1231792100 12810959 PMC164686

[35] Moradali MF, Donati I, Sims IM, Ghods S, Rehm BHA. 2015. Alginate polymerization and modification are linked in Pseudomonas aeruginosa. mBio 6:e00453-15. doi:10.1128/mBio.00453-15 25968647 PMC4436075

[36] Franklin MJ, Ohman DE. 2002. Mutant analysis and cellular localization of the AlgI, AlgJ, and AlgF proteins required for O acetylation of alginate in Pseudomonas aeruginosa. J Bacteriol 184:3000–3007. doi:10.1128/JB.184.11.3000-3007.2002 12003941 PMC135050

[37] Hestrin S. 1949. The reaction of acetylcholine and other carboxylic acid derivatives with hydroxylamine, and its analytical application. J Biol Chem 180:249–261. doi:10.1016/S0021-9258(18)56740-5 18133390

[38] Ciofu O, Tolker-Nielsen T. 2019. Tolerance and resistance of Pseudomonas aeruginosa biofilms to antimicrobial agents—how P. aeruginosa can escape antibiotics. Front Microbiol 10:913. doi:10.3389/fmicb.2019.00913 31130925 PMC6509751

[39] Tolker-Nielsen T. 2015. Biofilm development. Microbiol Spectr 3:MB–0001 doi:10.1128/microbiolspec.MB-0001-2014 26104692

[40] Ertesvåg H. 2015. Alginate-modifying enzymes: biological roles and biotechnological uses. Front Microbiol 6:523. doi:10.3389/fmicb.2015.00523 26074905 PMC4444821

[41] Lamppa JW, Griswold KE. 2013. Alginate lyase exhibits catalysis-independent biofilm dispersion and antibiotic synergy. Antimicrob Agents Chemother 57:137–145. doi:10.1128/AAC.01789-12 23070175 PMC3535929

[42] Abka-Khajouei R, Tounsi L, Shahabi N, Patel AK, Abdelkafi S, Michaud P. 2022. Structures, properties and applications of alginates. Mar Drugs 20:364. doi:10.3390/md20060364 35736167 PMC9225620

[43] Jones CJ, Wozniak DJ. 2017. Psl produced by mucoid Pseudomonas aeruginosa contributes to the establishment of biofilms and immune evasion. MBio 8:e00864-17. doi:10.1128/mBio.00864-17 28634241 PMC5478896

[44] Yang L, Hengzhuang W, Wu H, Damkiaer S, Jochumsen N, Song Z, Givskov M, Høiby N, Molin S. 2012. Polysaccharides serve as scaffold of biofilms formed by mucoid Pseudomonas aeruginosa. FEMS Immunol Med Microbiol 65:366–376. doi:10.1111/j.1574-695X.2012.00936.x 22309122

[45] Jacobs HM, O’Neal L, Lopatto E, Wozniak DJ, Bjarnsholt T, Parsek MR. 2022. Mucoid Pseudomonas aeruginosa can produce calcium-gelled biofilms independent of the matrix components Psl and CdrA. J Bacteriol 204:e0056821. doi:10.1128/jb.00568-21 35416688 PMC9112934

[46] Weissbach A, Hurwitz J. 1959. The formation of 2-keto-3-deoxyheptonic acid in extracts of Escherichia coli B. I. Identification. J Biol Chem 234:705–709. doi:10.1016/S0021-9258(18)70158-0 13654246

[47] Amador CI, Stannius RO, Røder HL, Burmølle M. 2021. High-throughput screening alternative to crystal violet biofilm assay combining fluorescence quantification and imaging. J Microbiol Methods 190:106343. doi:10.1016/j.mimet.2021.106343 34619138

[48] Peeters E, Nelis HJ, Coenye T. 2008. Comparison of multiple methods for quantification of microbial biofilms grown in microtiter plates. J Microbiol Methods 72:157–165. doi:10.1016/j.mimet.2007.11.010 18155789

[49] Kragh KN, Alhede M, Kvich L, Bjarnsholt T. 2019. Into the well—A close look at the complex structures of a microtiter biofilm and the crystal violet assay. Biofilm 1:100006. doi:10.1016/j.bioflm.2019.100006 33447793 PMC7798451

[50] Ghadam P, Akhlaghi F, Ali AA. 2017. One-step purification and characterization of alginate lyase from a clinical Pseudomonas aeruginosa with destructive activity on bacterial biofilm. Iran J Basic Med Sci 20:467–473. doi:10.22038/IJBMS.2017.8668 28656080 PMC5478773

[51] Daboor SM, Raudonis R, Cohen A, Rohde JR, Cheng ZMB. 2019. Marine bacteria, a source for alginolytic enzyme to disrupt Pseudomonas aeruginosa biofilms. Mar Drugs 17:307. doi:10.3390/md17050307 31137680 PMC6562671

[52] Xiao L, Han F, Yang Z, Lu X, Yu W. 2006. A novel alginate lyase with high activity on acetylated alginate of Pseudomonas aeruginosa FRD1 from Pseudomonas sp. QD03. World J Microbiol Biotechnol 22:81–88. doi:10.1007/s11274-005-7713-4

[53] Baker P, Ricer T, Moynihan PJ, Kitova EN, Walvoort MTC, Little DJ, Whitney JC, Dawson K, Weadge JT, Robinson H, Ohman DE, Codée JDC, Klassen JS, Clarke AJ, Howell PL. 2014. P. aeruginosa SGNH hydrolase-like proteins AlgJ and AlgX have similar topology but separate and distinct roles in alginate acetylation. PLoS Pathog 10:e1004334. doi:10.1371/journal.ppat.1004334 25165982 PMC4148444

[54] Dunphy LJ, Kolling GL, Jenior ML, Carroll J, Attai AE, Farnoud F, Mathers AJ, Hughes MA, Papin JA. 2021. Multidimensional clinical surveillance of Pseudomonas aeruginosa reveals complex relationships between isolate source, morphology, and antimicrobial resistance. mSphere 6:e0039321. doi:10.1128/mSphere.00393-21 34259555 PMC8386403

[55] Islam MM, Kolling GL, Glass EM, Goldberg JB, Papin JA. 2024. Model-driven characterization of functional diversity of Pseudomonas aeruginosa clinical isolates with broadly representative phenotypes. Microb Genom 10:001259. doi:10.1099/mgen.0.001259 38836744 PMC11261902

[56] Hentzer M, Teitzel GM, Balzer GJ, Heydorn A, Molin S, Givskov M, Parsek MR. 2001. Alginate overproduction affects Pseudomonas aeruginosa biofilm structure and function. J Bacteriol 183:5395–5401. doi:10.1128/JB.183.18.5395-5401.2001 11514525 PMC95424

[57] Candido Caçador N, Paulino da Costa Capizzani C, Gomes Monteiro Marin Torres LA, Galetti R, Ciofu O, da Costa Darini AL, Høiby N. 2018. Adaptation of Pseudomonas aeruginosa to the chronic phenotype by mutations in the algTmucABD operon in isolates from Brazilian cystic fibrosis patients. PLoS One 13:e0208013. doi:10.1371/journal.pone.0208013 30496246 PMC6264809

[58] Al Ahmar R, Kirby BD, Yu HD. 2020. Culture of small colony variant of Pseudomonas aeruginosa and quantitation of its alginate. J Vis Exp 60466. doi:10.3791/60466 32150164

[59] Valentine ME, Kirby BD, Withers TR, Johnson SL, Long TE, Hao Y, Lam JS, Niles RM, Yu HD. 2020. Generation of a highly attenuated strain of Pseudomonas aeruginosa for commercial production of alginate. Microb Biotechnol 13:162–175. doi:10.1111/1751-7915.13411 31006977 PMC6922527

[60] Frazier SB, Roodhouse KA, Hourcade DE, Zhang L. 2008. The quantification of glycosaminoglycans: a comparison of HPLC, carbazole, and Alcian Blue methods. Open Glycosci 1:31–39. doi:10.2174/1875398100801010031 20640171 PMC2904615

[61] Maccari F, Volpi N. 2022. Uronic acid carbazole assay and cetylpyridinium chloride titration depend on the chondroitin sulfate molecular weight. Anal Biochem 655:114848. doi:10.1016/j.ab.2022.114848 35948059

[62] Xie Y, He Y, Irwin PL, Jin T, Shi X. 2011. Antibacterial activity and mechanism of action of zinc oxide nanoparticles against Campylobacter jejuni. Appl Environ Microbiol 77:2325–2331. doi:10.1128/AEM.02149-10 21296935 PMC3067441

[63] Santander J, Martin T, Loh A, Pohlenz C, Gatlin DM, Curtiss R. 2013. Mechanisms of intrinsic resistance to antimicrobial peptides of Edwardsiella ictaluri and its influence on fish gut inflammation and virulence. Microbiology (Reading) 159:1471–1486. doi:10.1099/mic.0.066639-0 23676433 PMC4085987

[64] Riley LM, Weadge JT, Baker P, Robinson H, Codée JDC, Tipton PA, Ohman DE, Howell PL. 2013. Structural and functional characterization of Pseudomonas aeruginosa AlgX. J Biol Chem 288:22299–22314. doi:10.1074/jbc.M113.484931 23779107 PMC3829321

