# Supplementary material for: Applying a polysaccharide lyase from Stenotrophomonas maltophilia to disrupt alginate exopolysaccharide produced by Pseudomonas aeruginosa clinical isolates
Source: Appl Environ Microbiol. 2024 Dec 13;91(1):e01853-24. doi: 10.1128/aem.01853-24 (PMC11784403; doi:10.1128/aem.01853-24)
Supplement: Supplemental figures — Figures S1 to S4. [file aem.01853-24-s0001.pdf]

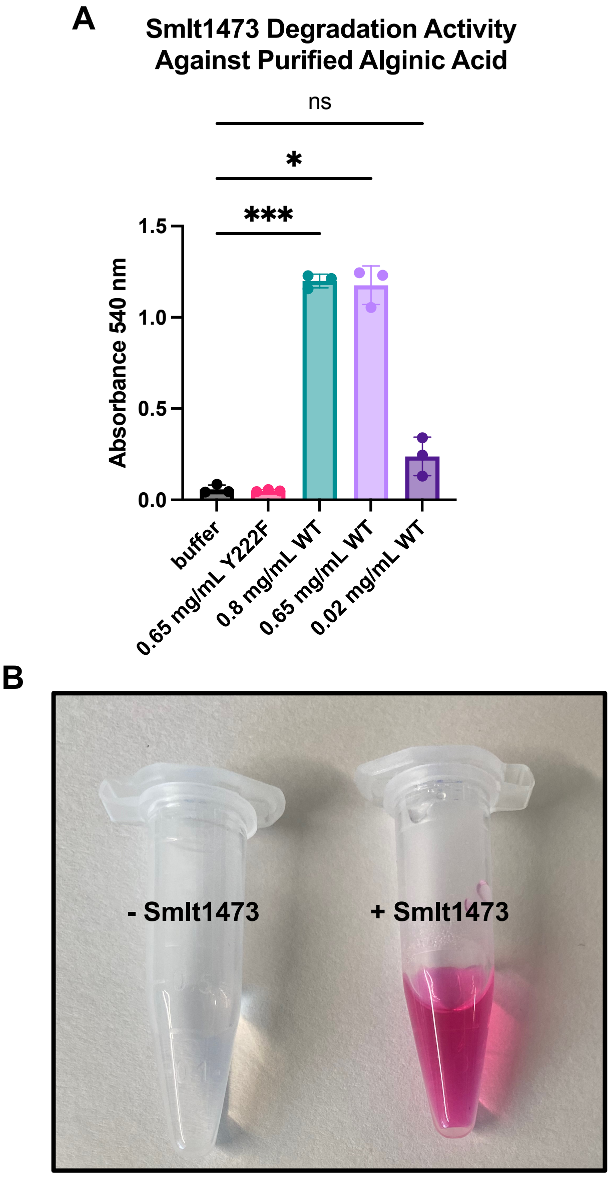

Figure S1. Smlt1473 displays degradation activity against purified alginic acid. (A) Depolymerization of alginic acid by various concentrations of Smlt1473 was detected by the TBA assay. (B) In the presence of Smlt1473, alginate depolymerization is visually depicted by the appearance of a pink chromogen with an absorbance at 540 nm, whereas in the absence of Smlt1473, the solution remains clear.

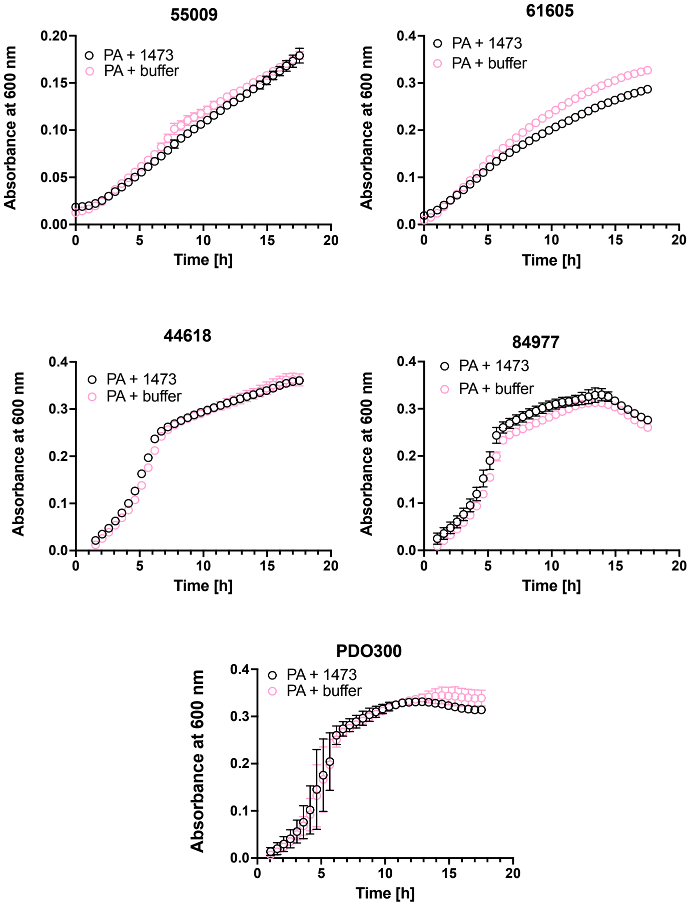

Figure S2. Smlt1473 does not inhibit growth of *P. aeruginosa*. Growth curves for each *P. aeruginosa* isolate in the presence and absence of Smlt1473 are shown. A concentration of 0.47 mg/mL Smlt1473 was used as it was the highest achievable concentration in liquid culture. Growth was monitored at 37 °C for 17.5 h. Each isolate was run in triplicate.

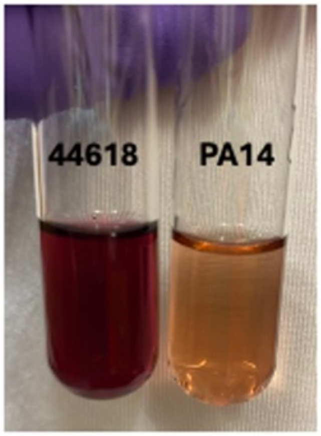

Figure S3. Carbazole assay shows specificity for uronic acid. (A) The carbazole assay was conducted using supernatant from a mucoid, alginate producing clinical isolate of *P. aeruginosa* (UVA 44618) and supernatant from a nonmucoid strain (PA14), where alginate is not a significant component of the EPS matrix. When uronic acid from alginate is present (UVA 44618), there is a deep purple color observed. When uronic acid content is low (PA14), there is significantly less color reactivity.

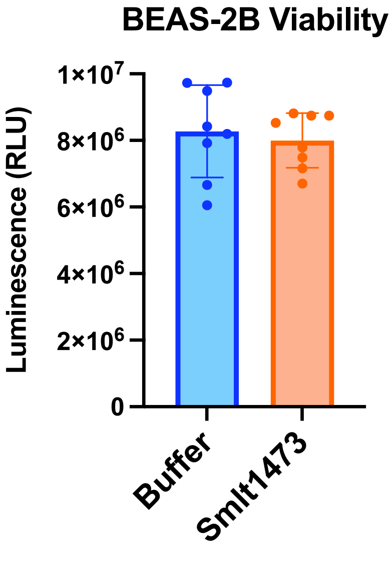

Figure S4. Smlt1473 is non-cytotoxic to bronchial epithelial cells. BEAS-2B cells were grown and maintained according to ATCC protocols. Cells were seeded in a 96-well plate at 10,000 cells per 100 mL and incubated at 37 °C and 5% CO2 overnight to allow for attachment. Media was removed and fresh was added containing either buffer or 0.2 mg/mL Smlt1473. CellTiter-Glo Luminescent Cell Viability Assay (Promega) was conducted and showed that Smlt1473 is non-cytotoxic to BEAS-2B cells.
